# The Association between Chronic Kidney Disease and Diabetic Retinopathy: The Korea National Health and Nutrition Examination Survey 2008-2010

**DOI:** 10.1371/journal.pone.0125338

**Published:** 2015-04-07

**Authors:** Young-Hoon Park, Jeong Ah Shin, Jae-Hyung Han, Yong-Moon Park, Hyeon Woo Yim

**Affiliations:** 1 Department of Ophthalmology and Visual Science, College of Medicine, The Catholic University of Korea, Seoul, Korea; 2 Department of Preventive Medicine, College of Medicine, The Catholic University of Korea, Seoul, Korea; 3 Department of Epidemiology and Biostatistics, Arnold School of Public Health, University of South Carolina, Columbia, SC, United States of America; University of Glasgow, UNITED KINGDOM

## Abstract

**Purpose:**

To explore the relationship between chronic kidney disease and diabetic retinopathy in a representative population of Korean diabetic adults.

**Methods:**

We analyzed data from the Korea National Health and Nutrition Examination Surveys (2008-2010). A total of 15,409 individuals (weighted frequency, 32,168,636) aged 19 and over who completed ophthalmologic and renal functional examinations were evaluated. Chronic kidney disease (CKD) was defined as an estimated glomerular filtration rate of < 60 ml/min/1.73 m^2^ or proteinuria greater than 1+. Seven standard photographs from the Early Treatment for Diabetic Retinopathy Study were obtained from each eye after pharmacological pupil dilatation. Diabetic retinopathy (DR) was defined as the presence of 1 or more retinal microaneurysms or retinal blot hemorrhages with or without more severe lesions. Vision-threatening diabetic retinopathy (VTDR) was defined as the presence of a clinically significant macular edema (CSME) or proliferative diabetic retinopathy.

**Results:**

CKD was significantly associated with DR and VTDR (odds ratio (OR), 95% confidence interval (CI); 2.49(1.43-4.35) and 3.74(1.56-8.95), respectively) in the diabetic population. After controlling for confounders, however, CKD was significantly associated only with DR [adjusted OR (aOR), 95% CI; 2.34(1.04-5.28)]. In the subgroup analysis for CKD, only proteinuria was significantly associated with DR and VTDR (aOR, 95% CI; 4.56(1.51-13.77) and 5.61(1.06-29.87), respectively) in this population.

**Conclusions:**

Our results show that CKD appears to be associated with DR and VTDR in a Korean diabetic population. In particular, proteinuria, not decreased eGFR, is more significantly associated with DR or VTDR.

## Introduction

Diabetes mellitus (DM) is one of the most important and common chronic diseases worldwide and is expected to increase in prevalence due to population growth, ageing, and escalating rates of obesity [1,2]. The prevention of DM complications is important because the morbidity, mortality, and health care costs for diabetic patients is a critical socioeconomic issue in most countries [3]. Diabetic retinopathy (DR) is the most common cause of visual disability in people of working age [4]. It is well known that the risk factors of DR include the duration of DM, blood pressure, poor glycemic control, and obesity [5,6].

Chronic kidney disease (CKD), another serious worldwide health problem associated with cardiovascular and renal complications, is also rapidly increasing in prevalence [7,8].

Interestingly, DR and CKD are closely associated with age and metabolic and cardiovascular risk factors, such as hypertension, DM, obesity and hyperlipidemia [5, 9–13]. Retinal microvascular signs, such as retinopathy and venular dilatation, have been shown to be predictive of CKD development, and patients with CKD may be at higher risk for many eye diseases including DR [14,15]. Some studies have documented the associations between albuminuria and retinopathy in individuals with DM [16,17]. Both microalbuminuria and gross proteinuria were reported to be associated with DR in a population-based study of diabetes-related complications and their risk factors [18]. Although recent few reports have suggested that CKD is associated with DR, this relationship has not been properly investigated in Asian diabetic patients.

The aim of the present study was to investigate the association of CKD and DR and VTDR in a representative Korean diabetic population.

## Methods

### Study population

The Korea National Health and Nutrition Examination Survey (KNHANES) is an ongoing cross-sectional survey for the non-institutionalized civilian population of South Korea. A complex, stratified, multistage probability sampling design based on age, sex, and region was used in this survey to represent the Korean population. Since KNHANES IV, a rolling sampling design also has been used so that the samples from each year are independent and homogeneous. KNHANES, coordinated by the Korean Ministry of Health and Welfare, included health interview, health examination, and nutrition surveys. Since 2008, ophthalmologic interviews and examinations have also been conducted. This study was reviewed and approved by the Institutional Review Boards of the Seoul St. Mary’s Hospital College of Medicine the Catholic University of Korea (IRB #, KC14EISI0686), and all participants provided written informed consent.

DM was diagnosed on the basis of a fasting plasma glucose levels >126 mg/dL, self-reported diagnosed DM, or current use of oral hypoglycemic agents and/or insulin according to the clinical practice guidelines from the Korea Diabetes Association [19]. We considered that participants have type 1 DM if they were younger than 30 years old at the time DM diagnosis and were receiving insulin therapy. In this study, we analyzed the total DM participant aged 19 and over, including type 1 and type 2 DM.

In the present analysis, we limited the study population to diabetic adults aged 19 years or older who participated in all three parts of the survey in addition to ophthalmologic interviews and examinations.

### Assessment of DR

Ophthalmologic examinations were conducted by ophthalmologists from the Korean Ophthalmologic Society in cooperation with the Korea Centers for Disease Control and Prevention (KCDC). Participants underwent a comprehensive eye slit-lamp examination (Haag-Streit BQ-900; Haag-Streit AG, Koeniz, Switzerland) by ophthalmologists.

Seven standard photographs from the Early Treatment for Diabetic Retinopathy Study were obtained from each eye after pharmacological pupil dilatation. Diabetic retinopathy (DR) was defined as the presence of 1 or more retinal microaneurysms or retinal blot hemorrhages with or without more severe lesions (hard exudates, soft exudates, intraretinal microvascular abnormalities, venous bleeding, new retinal vessels, and fibroproliferations). Vision-threatening diabetic retinopathy (VTDR) was defined as the presence of clinically significant macular edema (CSME) or proliferative diabetic retinopathy. The final retinopathy grading for each participant was based on the diagnosis in the more severely affected eye. Two ophthalmologists reviewed all the files.

### Assessment of CKD

Chronic kidney disease (CKD) was defined as an estimated glomerular filtration rate (eGFR) of < 60 ml/min/1.73 m^2^ or proteinuria greater than 1+. The GFR was estimated from serum creatinine concentrations using the Modification of Diet in Renal Disease (MDRD) equation defined as the following: eGFR = 186.3 x (serum creatinine in mg/dl)-1.154 x age -0.203 (x 0.742 for women). Serum creatinine was measured using a Hitachi Automatic Analyzer 7600 (Hitachi, Japan) by the standardized Jaffe compensated kinetic method; urine protein was measured using the dipstick test, and results are reported on a semiquantitative scale from negative to positive four.

### Measurements

The health interview survey was performed by trained interviewers. All participants were questioned about their demographic and socioeconomic characteristics, including residential area, education, income, and occupation. Ophthalmologic surveys were also conducted. Respondents were categorized into two groups: ever-smokers (current smokers and ex-smokers) and non-smokers. Alcohol consumption was determined by questioning the participants about their drinking behaviors during the month before the health interview. After converting the average frequency and amount of alcoholic beverages into the amount of pure alcohol (in grams) consumed per day, we classified respondents on the basis of alcohol consumption into two groups: non- or moderate drinkers (< 30.0 g alcohol/day) and heavy drinkers (≥ 30.0 g alcohol/day) [20]. The subjects who engaged in moderate or vigorous exercise on a regular basis were designated as those who exercised regularly.

Anthropometric measurements of the participants were performed by specially trained examiners. Waist circumference was measured to the nearest 0.1 cm on a horizontal plane at the midpoint between the iliac crest and the costal margin at the end of a normal expiration. The body mass index (BMI) was calculated as the patient’s weight in kilograms divided by the square of the patient’s height in meters. Blood pressure was measured three times on the right arm while the individual was in a seated position after at least 5 minutes of rest using a mercury sphygmomanometer (Baumanometer; Baum, Copiague, NY). The final blood pressure value was obtained by averaging the values of the second and third blood pressure measurements.

Blood samples were obtained after a minimum fasting period of 8 hours. The serum levels of glucose, total cholesterol (TC), high-density lipoprotein (HDL) cholesterol, triglycerides, creatinine, asparate aminotransferase (AST), and alanine aminotransferase (ALT) were measured enzymatically using a Hitachi automatic analyzer 7600 (Tokyo, Japan). Hemoglobin (Hb) was measured by the SLS hemoglobin (NoCyanide) method using an XE-2100D (Sysmex/Japan). Insulin resistance was calculated using the homeostasis model assessment (HOMA) estimate of insulin resistance (HOMA-IR = fasting insulin [uU/ml] x fasting glucose [mmol/l]/22.5) [21].

### Definition of Metabolic Syndrome (MetS)

MetS was defined using the criteria proposed by the American Heart Association (AHA) and the National Heart, Lung, and Blood Institute (NHLBI) together with the International Diabetes Federation (IDF) in 2009 [33]. MetS was defined as (1) a waist circumference ≥ 90 cm in men and ≥ 80 cm in women according to the IDF criteria for Asian countries; (2) a fasting glucose ≥ 100 mg/dl or usage of medication to control elevated glucose; (3) fasting triglycerides ≥ 150 mg/dl or usage of cholesterol-lowering medication; (4) HDL-cholesterol < 40 mg/dL in men and < 50 mg/dL in women or usage of cholesterol-lowering medication; and (5) systolic blood pressure ≥ 130 mmHg and/or diastolic blood pressure ≥ 85 mm Hg or using an antihypertensive drug treatment for patients with a history of hypertension. MetS diagnosis required at least three of the five components to be present.

#### Statistical analysis

All data are presented as the mean ± SE for continuous variables or proportions for categorical variables. Statistical analyses were conducted using the SAS (version 9.3; SAS Institute, Inc., Cary, NC, USA) survey procedure to take into account the complex sampling design with sampling weights of KNHANES and to provide nationally representative prevalence estimates. In order to minimize the effect of variations among the survey years, all the analyses performed in this study were adjusted for survey year. In addition, we conducted analyses to assess the effects of DM duration and DM control in the association between CKD and DR. Multiple logistic regression analyses were performed to estimate the magnitude of the association between DR and CKD and its components, and two statistical models based on the characteristics of the variables were used. One model included age and sex. Subsequently, socioeconomic and lifestyle-related characteristics, including education, smoking status, drinking alcohol, obesity, and MetS components, were included based on the results from the univariate analysis. A P < 0.05 was considered statistically significant.

## Results

### 1. The prevalence and related characteristics of chronic kidney disease

The prevalence of CKD in this diabetic population was 10.62% (SE, 0.95%; 11.31% for men and 9.80% for women). The prevalence of individual CKD components was 7.30% for eGFR<60 ml/min/1.73 m^2^ and 5.03% for proteinuria in the diabetic population. Table 1 shows the characteristics of the participants by CKD status. Subjects with CKD were more likely to be older than those without CKD. Regular exercise and higher education were lower, whereas obese, lower HDL, higher blood pressure, and longer DM duration were higher in participants with CKD.

**Table 1:** Characteristics of the participants according to the presence or absence of chronic kidney disease in a diabetic population.

	CKD		*P*	eGFR< 60		*P*	Proteinuria		*P*
	absence	presence		absence	presence		absence	presence	
Sex (%)			0.408			0.833			0.008
male	54.2(1.8)	58.2(4.2)		54.7(1.7)	53.6(5.1)		55.0(1.7)	72.8(5.7)	
female	45.8(1.8)	41.8(4.2)		45.3(1.7)	46.4(5.1)		45.0(1.7)	27.2(5.7)	
Age (years)			<.001						
19–29	1.3(0.6)	1.3(1.3)							
30–39	6.4(0.9)	4.4(2.0)							
40–49	20.4(1.7)	5.8(2.9)							
50–59	28.0(1.6)	19.3(3.4)							
60–69	26.3(1.4)	28.2(3.9)							
≥70	17.6(1.3)	41.0(4.5)							
Education (> 6 yrs)	60.6(1.9)	49.9(4.5)	0.022	60.8(1.8)	42.6(5.1)	<.001	58.9(1.8)	69.4(6.0)	0.110
Ever-smoker (yes)	48.6(1.7)	48.9(4.3)	0.945	48.5(1.7)	50.4(4.7)	0.693	49.0(1.7)	53.4(7.5)	0.572
Heavy drinker (yes)	20.9(1.9)	15.8(4.4)	0.316	20.7(1.9)	16.0(5.5)	0.444	20.7(1.9)	19.6(7.5)	0.889
Regular exercise (yes)	26.2(1.6)	13.9(2.7)	<.001	25.9(1.6)	12.5(3.0)	0.001	25.6(1.5)	13.1(3.8)	0.012
MI (> = 25 kg/m^2^)	49.8(1.8)	52.8(4.6)	0.014	50.3(1.7)	47.8(4.9)	0.181	49.2(1.8)	60.3(7.3)	0.121
MetS components									
High WC	56.3(1.8)	57.1(4.4)	0.860	56.1(1.8)	59.8(4.8)	0.472	56.0(1.8)	53.7(7.3)	0.766
Low HDL	62.3(1.7)	74.9(4.2)	0.009	62.3(1.7)	80.1(4.1)	<.001	62.6(1.7)	72.2(7.0)	0.203
High glucose	89.1(1.0)	83.6(3.0)	0.058	89.3(1.0)	77.8(4.0)	<.001	88.4(1.0)	90.3(4.0)	0.678
High triglycerides	55.5(1.7)	60.0(4.4)	0.347	55.5(1.7)	61.7(4.7)	0.224	55.4(1.7)	60.0(7.5)	0.563
High blood pressure	61.5(1.9)	82.3(3.3)	<.001	62.2(1.8)	83.0(3.8)	<.001	62.8(1.7)	84.5(4.6)	<.001
Systolic blood pressure (mmHg)	124.4(0.6)	128.9(1.6)	0.009	124.6(0.6)	128.5(1.9)	0.053	124.5(0.6)	131.6(2.2)	0.002
Diastolic blood pressure (mmHg)	77.1(0.4)	75.3(1.0)	0.104	77.2(0.4)	72.9(1.1)	<.001	76.9(0.3)	79.6(1.4)	0.051
DM duration (> = 5 yr)	57.5(2.2)	80.6(4.2)	<.001	58.0(2.1)	84.0(4.1)	<.001	59.4(2.1)	79.3(7.4)	0.031
DM control (yes)	29.2(1.8)	32.7(4.3)	0.445	29.1(1.7)	35.6(4.7)	0.188	29.4(1.7)	25.0(7.0)	0.333
HbA1c	7.3(0.1)	7.4(0.1)	0.582	7.3(0.1)	7.3(0.2)	0.753	7.3(0.1)	7.7(0.2)	0.162
DMR			<.001			0.025			<.001
NPDR	15.6(1.8)	29.6(6.1)		16.4(1.8)	24.9(6.6)		16.3(1.8)	37.5(10.3)	
PDR	1.8(0.5)	5.5(2.8)		1.8(0.5)	7.1(4.0)		1.8(0.5)	4.9(3.7)	
CSME	1.4(0.5)	6.4(2.7)		1.7(0.5)	4.1(3.0)		1.4(0.5)	12.8(6.0)	

Data are presented as the mean±SE or % (SE). Abbreviation: CKD, chronic kidney disease; DM, diabetes mellitus; BMI, body mass index; MetS, metabolic syndrome; WC, waist circumference; HDL, high density lipoprotein; NPDR, non-proliferative diabetic retinopathy; PDR, proliferative diabetic retinopathy; CSME, clinically significant macular edema. DM control means HbA1c<6.5.

### 2. The prevalence and related characteristics of diabetic retinopathy

The mean (SE) of DM duration in this population was 7.59 (0.27) years. The prevalence of DR in this diabetic population was 11.39% (SE, 1.06%; 9.99% for men and 13.08% for women). The prevalence of VTDR in this population was 2.20% (SE, 0.39%; 1.95% for men and 2.50% for women). Table shows the characteristics of the participants by DR or VTDR status (S1 Table). Subjects with DR or VTDR were more likely to be thinner in the Korean DM population. A longer duration of DM and poor DM control were higher in participants with VTDR.

### 3. The associations between chronic kidney disease and diabetic retinopathy

Table 2 shows the results of multiple logistic regression analyses. After controlling for confounders, CKD was significantly associated with DR (adjusted odds ratio (aOR), 2.34; 95% confidence interval (CI), 1.04–5.28) but was not associated with VTDR. However, proteinuria was significantly associated with DR and VTDR (aOR, 4.56 and 5.61; 95% CI, 1.51–13.77 and 1.06–29.87, respectively).

**Table 2:** Association of chronic kidney disease/ proteinuria with diabetic retinopathy/ vision-threatening diabetic retinopathy in a diabetic population.

Variables	Diabetic retinopathy				Vision-threatening DR			
	Model 1		Model 2		Model 1		Model 2	
	OR(95% CI)	*P*	OR(95% CI)	*P*	OR(95% CI)	*P*	OR(95% CI)	*P*
CKD	2.49(1.43,4.35)	0.001	2.34(1.04,5.28)	0.041	3.74(1.56,8.95)	0.003	2.08(0.50,8.63)	0.314
eGFR<60 ml/min/1.73 m^2^	1.94(1.01,3.72)	0.046	1.50(0.50,4.47)	0.471	3.15(1.00,9.90)	0.049	1.16(0.17,8.15)	0.879
Proteinuria	3.28(1.63,6.60)	<.001	4.56(1.51,13.77)	0.007	5.52(2.12,14.36)	<.001	5.61(1.06,29.87)	0.043

Data are presented as the mean±SE or % (SE). Abbreviation: DR, diabetic retinopathy; VTDR, vision-threatening DR; DM, diabetes mellitus. Model 1: adjusted by age and sex; model 2: adjusted by age, sex, education, smoking status, drinking alcohol, exercise, obesity (BMI), metabolic syndrome components (high waist circumference, low HDL, high glucose, high triglycerides, high blood pressure), DM duration, and HbA1c level.

### 4. The associations between subgroups of chronic kidney disease and diabetic retinopathy

Table 3 shows the results of multiple logistic regression analyses. After controlling for confounders, decreased GFR (eGFR<60 ml/min/1.73 m^2^) with proteinuria significantly associated with VTDR (aOR, 28.81; 95% CI, 1.19–696.6).

**Table 3:** Association of the subgroups of chronic kidney disease with diabetic retinopathy/ vision-threatening diabetic retinopathy in a diabetic population.

Variables	Diabetic retinopathy				Vision-threatening DR			
	Model 1		Model 2		Model 1		Model 2		
	OR(95% CI)	*P*	OR(95% CI)	*P*	OR(95% CI)	*P*	OR(95% CI)	*P*
eGFR<60 ml/min/1.73 m^2^								
Proteinuria	2.20(0.62,7.75)	0.222	4.68(0.44,50.02)	0.202	10.16(1.52,68.03)	0.017	28.81(1.19,696.61)	0.039
Non- proteinuria	0.46(0.13,1.61)	0.222	0.21(0.02,2.28)	0.202	0.10(0.02,0.66)	0.017	0.04(0.001,0.84)	0.039

Data are presented as the mean±SE or % (SE). Abbreviation: DR, diabetic retinopathy; VTDR, vision-threatening DR; DM, diabetes mellitus. Model 1: adjusted by age and sex; model 2: adjusted by age, sex, education, smoking status, drinking alcohol, exercise, obesity (BMI), metabolic syndrome components (high waist circumference, low HDL, high glucose, high triglycerides, high blood pressure), DM duration, and HbA1c level.

## Discussion

To the best of our knowledge, this is the first large population based study to examine the association between DR and CKD and its components (decreased eGFR and proteinuria) among a representative Korean diabetic population. In this cross-sectional study, conducted as part of the 2008–2010 KNHANES, CKD and its components, including proteinuria, were positively associated with the risk of DR and VTDR.

In this study, we found that the prevalence of CKD in this diabetic population was 10.62%. Recent studies show a worldwide increase in CKD [7,11,22,23]. Generally, approximately 10 to 15% of the adults suffer from CKD: 13.1% in the US, 12.9% in Japan, and 13.0% in China [7,11,22,23]. The prevalence of CKD in Korean adults seems to be lower than in adult Western and East Asian populations [24]. However, because the duration and control of diseases have significant differences among these studies. Simple and direct comparison has some limitations.

Another Korean national data set shows that the prevalence of CKD in KNHANES IV was 4.5% in men and 6.3% in women [25]. However, because DM is one of the most important risk factors, the prevalence of CKD in the DM population seems to be higher, as observed in our data.

The prevalence of individual CKD components was 7.30% for eGFR<60 ml/min/1.73 m^2^ and 5.03% for proteinuria in the diabetic population. Subjects with CKD were more likely to be older. Regular exercise and higher education were lower, whereas obesity, lower HDL, higher blood pressure, and longer DM duration were more frequent in participants with DM.

Recently, one national cross-sectional study that performed in Spanish primary care centers reported that the prevalence was 18.0% for eGFR<60 ml/min/1.73 m^2^ and 15.4% for albuminuria [26]. A study performed in Japan reported that 25.2% of type 2 DM patients had a GFR below 60 ml/min/1.73 m^2^ [27]. Different prevalence rates of CKD among these studies may due to the different methodology applied and the racial differences among patients. In addition, the decreased prevalence of CKD in Korean adults in a recent survey can be partly explained by the combined effect of not only active management of chronic diseases such as hypertension, diabetes and dyslipidemia, but also the increased number of subjects who were regularly engaged in exercise [25].

The Kidney Disease Outcomes Quality Initiative guideline defines CKD as the presence of either renal dysfunction (GFR <60 mL/min/1.73 m^2^) or renal damage (proteinuria or albuminuria) lasting for ≥3 months [28]. However, we defined CKD using a single measurement of the estimated GFR based on the MDRD equation and of proteinuria by a dipstick test.

In the present study, the prevalence of DR in this diabetic population was 11.39% (SE, 1.06%; 9.99% for men and 13.08% for women). The prevalence of VTDR in this population was 2.20% (SE, 0.39%; 1.95% for men and 2.50% for women). Previously reported studies have shown considerable variability in DR prevalence rates among individuals with DM, with rates ranging from 17.6% to 33.2% [29,30]. Differences in study methodologies, population characteristics, and ascertainment and classification of DR have made direct comparisons between studies difficult. Recently, Yau et al.[5] reported in a systematic literature review that the overall global prevalence was 34.6% for any DR, 6.96% for proliferative DR, and 10.2% for VTDR. In addition, they concluded that the prevalence estimates of any DR and VTDR were highest in African Americans and lowest in Asians as in our data [5]. Differences in the prevalence of DR among ethnic groups have also been reported [31,32].

CKD was significantly associated with DR (aOR, 2.34; 95% CI, 1.04–5.28) but was not associated with VTDR. However, insufficient statistical power due to a small sample size of incident VTDR cases may explain the lack of association between CKD and VTDR. Therefore we are unable to confidently conclude that presence of CKD was associated with increased risk of VDTR development in participants with DM in the current study. Further studies will be needed to confirm this exploratory finding. Proteinuria was significantly associated with DR and VTDR (aOR, 4.56 and 5.61; 95% CI, 1.51–13.77 and 1.06–29.87, respectively). Proteinuria seems to be a more important indicator for DR and VTDR than decreased eGFR.

Penno et al.[33] reported that CKD was present in 58.64% of subjects with advanced DR, whereas advanced DR was detectable only in 15.28% of individuals with any CKD and correlated with albuminuric CKD phenotypes more than with nonalbuminuric phenotype. These findings were consistent with our data, but their study was hospital-based cohort study and had a limitation of only using the fundoscopic findings instead of the reference method for DR diagnosis.

Our study should be interpreted with consideration of the following limitations. First, this study was a cross-sectional analysis; therefore, causal relationships could not be identified, nor were the mechanisms of these associations explored. Second, differences in thresholds might arise when the analysis is based on any DR as opposed to diabetes-specific DR. In other words, a microaneurysm could be caused by other pathologies besides diabetes. Third, we analyzed total DM participants including type 1 and type 2 DM together. However, because the number of type 1 DM was too small (only 6 participants), and the number were statistically insignificant, we have included and analyzed DM type I and II participants aged 19 and over. Actually the statistical results were same using only the data of type 2 DM participants. Fourth, using proteinuria greater 1+ is a limitation. It does not adequately investigate the relationship of DR with urinary protein excretion, but in this version of cross-sectional study, we could not get more information from the urine analysis (urine albumin level, etc.). Fifth, we could not get the data of drugs, which could affect the assessment of CKD and proteinuria as ACE inhibitors or angiotensin II receptor blockers. Despite these limitations, this study used a nationally representative sample of adults in Korea, which is a crucial strength. Additionally, we assessed all of the DM population 19 years and older. Moreover, to the best of our knowledge, this is the first large population based study to examine the association between DR and CKD and its components (decreased eGFR and proteinuria) among a representative Korean diabetic population.

Taken together, CKD appear to be associated with DR and VTDR in a Korean DM population. In particular, proteinuria, not decreased eGFR, is more significantly associated with DR or VTDR in diabetic patients.

## Supporting Information

S1 Table Characteristics of the participants according to the presence or absence of diabetic retinopathy or vision-threatening diabetic retinopathy in a diabetic population. (DOCX)
