# Supplementary material for: The Association between Chronic Kidney Disease and Diabetic Retinopathy: The Korea National Health and Nutrition Examination Survey 2008-2010
Source: PLoS One. 2015 Apr 7;10(4):e0125338. doi: 10.1371/journal.pone.0125338 (PMC4388494; doi:10.1371/journal.pone.0125338)
Supplement: S1 Table — (DOCX) [file pone.0125338.s001.docx]

Table 1. Characteristics of the participants according to the presence or absence of diabetic retinopathy or vision-threatening diabetic retinopathy in a diabetic population

|  | Diabetic retinopathy | | *P* |  | Vision-threatening DR | | *P* |
| --- | --- | --- | --- | --- | --- | --- | --- |
|  | absence | presence |  |  | absence | presence |  |
| Sex (%) |  |  | 0.146 |  |  |  | 0.502 |
| male | 55.5(1.7) | 47.9(5.1) |  |  | 54.8(1.7) | 48.4(9.2) |  |
| female | 44.5(1.7) | 52.1(5.1) |  |  | 45.2(1.7) | 51.6(9.2) |  |
| Age (years) |  |  | 0.752 |  |  |  | NA |
| 19-29 | 1.3(0.6) | 1.6(1.3) |  |  | 1.3(0.5) | 0.0(0.0) |  |
| 30-39 | 5.9(0.8) | 8.2(3.0) |  |  | 6.2(0.8) | 1.4(1.4) |  |
| 40-49 | 19.2(1.7) | 16.1(4.1) |  |  | 18.9(1.6) | 15.8(6.4) |  |
| 50-59 | 27.6(1.5) | 23.1(4.2) |  |  | 26.9(1.5) | 37.3(9.1) |  |
| 60-69 | 25.9(1.4) | 30.9(4.3) |  |  | 26.3(1.3) | 33.8(8.6) |  |
| ≥70 | 20.1(1.2) | 20.1(4.0) |  |  | 20.3(1.3) | 11.7(5.9) |  |
| Education (> 6 yrs) | 59.2(1.8) | 61.0(4.9) | 0.715 |  | 59.5(1.8) | 52.8(9.5) | 0.466 |
| Ever-smoker (yes) | 49.8(1.6) | 39.9(5.0) | 0.054 |  | 48.9(1.6) | 38.5(9.2) | 0.280 |
| Heavy drinker (yes) | 20.5(1.9) | 21.0(5.5) | 0.938 |  | 20.7(1.8) | 15.1(8.9) | 0.576 |
| Regular exercise (yes) | 25.6(1.6) | 19.8(3.9) | 0.199 |  | 24.9(1.5) | 27.1(8.9) | 0.801 |
| BMI (>= 25 kg/m^2^) | 51.6(1.8) | 40.3(4.9) | 0.021 |  | 50.7(1.7) | 31.6(7.9) | 0.025 |
| MetS components |  |  |  |  |  |  |  |
| High waist circumference | 42.6(1.7) | 48.3(5.2) | 0.082 |  | 56.7(1.7) | 43.4(9.3) | 0.159 |
| Low HDL | 62.8(1.7) | 71.0(4.8) | 0.121 |  | 63.4(1.6) | 77.2(7.0) | 0.086 |
| High glucose | 88.5(1.1) | 88.3(3.5) | 0.956 |  | 88.6(1.0) | 82.5(8.1) | 0.377 |
| High triglycerides | 54.8(1.7) | 63.6(4.9) | 0.101 |  | 55.7(1.6) | 61.5(9.1) | 0.528 |
| High blood pressure | 65.4(1.8) | 50.7(5.0) | 0.005 |  | 63.7(1.7) | 62.2(8.8) | 0.859 |
| DM duration (>= 5 yr) | 58.7(2.1) | 68.2(5.2) | 0.106 |  | 59.5(2.0) | 81.4(7.8) | 0.029 |
| DM control (yes) | 32.2(1.8) | 7.9(2.2) | <.001 |  | 29.8(1.6) | 12.6(6.0) | 0.043 |

Data are presented as the mean±SE or % (SE). Abbreviation: DR, diabetic retinopathy; VTDR, vision-threatening DR; DM, diabetes mellitus; BMI, body mass index; MetS, metabolic syndrome; HDL, high density lipoprotein. DM control means HbA1c<6.5.
